# Differential Cytotoxicity and Inflammatory Responses to Particulate Matter Components in Airway Structural Cells

**DOI:** 10.3390/ijms26020830

**Published:** 2025-01-20

**Authors:** Nilofar Faruqui, Sofie Orell, Camilla Dondi, Zaira Leni, Daniel M. Kalbermatter, Lina Gefors, Jenny Rissler, Konstantina Vasilatou, Ian S. Mudway, Monica Kåredal, Michael Shaw, Anna-Karin Larsson-Callerfelt

**Affiliations:** 1 Department of Chemical & Biological Services, National Physical Laboratory, Teddington TW11 0LW, UK; 2 Lung Biology, Department of Experimental Medical Sciences, Lund University, 221 84 Lund, Sweden; 3 Institute of Anatomy, University of Bern, 3012 Bern, Switzerland; 4 Federal Institute of Metrology METAS, CH-3003 Bern-Wabern, Switzerland; 5 Lund University Bioimaging Centre (LBIC), Lund University, 221 84 Lund, Sweden; 6 Ergonomics and Aerosol Technology, Department of Design Sciences, Faculty of Engineering (LTH), Lund University, 223 62 Lund, Sweden; 7 MRC Centre for Environment and Health, Imperial College London, London W2 1PG, UK; 8 National Institute of Health Protection Research Unit in Environmental Exposures and Health, London W2 1NY, UK; 9 Division of Occupational and Environmental Medicine, Department of Laboratory Medicine, Lund University, 223 63 Lund, Sweden; 10 Department of Occupational and Environmental Medicine, Region Skåne, 223 63 Lund, Sweden; 11 Department of Computer Science, University College London, London WC1E 6BT, UK

**Keywords:** particulate matter, elemental and organic carbon, copper oxide, ammonium salts, epithelial cells, lung fibroblasts, precision-cut lung slices

## Abstract

Particulate matter (PM) is a major component of ambient air pollution. PM exposure is linked to numerous adverse health effects, including chronic lung diseases. Air quality guidelines designed to regulate levels of ambient PM are currently based on the mass concentration of different particle sizes, independent of their origin and chemical composition. The objective of this study was to assess the relative hazardous effects of carbonaceous particles (soot), ammonium nitrate, ammonium sulfate, and copper oxide (CuO), which are standard components of ambient air, reflecting contributions from primary combustion, secondary inorganic constituents, and non-exhaust emissions (NEE) from vehicular traffic. Human epithelial cells representing bronchial (BEAS-2B) and alveolar locations (H441 and A549) in the airways, human lung fibroblasts (HFL-1), and rat precision-cut lung slices (PCLS) were exposed in submerged cultures to different concentrations of particles for 5–72 h. Following exposure, cell viability, metabolic activity, reactive oxygen species (ROS) formation, and inflammatory responses were analyzed. CuO and, to a lesser extent, soot reduced cell viability in a dose-dependent manner, increased ROS formation, and induced inflammatory responses. Ammonium nitrate and ammonium sulfate did not elicit any significant cytotoxic responses but induced immunomodulatory alterations at very high concentrations. Our findings demonstrate that secondary inorganic components of PM have a lower hazard cytotoxicity compared with combustion-derived and indicative NEE components, and alveolar epithelial cells are more sensitive to PM exposure. This information should help to inform which sources of PM to target and feed into improved, targeted air quality guidelines.

## 1. Background

Ambient air pollution is a major health problem globally, with particulate matter (PM) being one of the main air pollutants and a key driver of global morbidity and premature mortality. Epidemiological and toxicological studies indicate that there are strong associations and plausible causal pathways between exposure to PM and adverse health effects, including dementia, lung cancer, and cardiovascular and respiratory diseases [1,2,3]. PM is a complex mixture of microscopic, solid, and liquid particles containing acids, metals, organic chemicals (including carbonaceous organic matter), soil, and dust particles. The characteristics of PM, i.e., size, morphology, and chemical composition, differ with time and location, depending on the pollutant sources from which they originate and atmospheric processing/aging [4]. Particle size is a key factor in determining the site of deposition in the airways, which in turn affects clearance and induction of potential adverse health effects. For regulatory purposes, particles are commonly divided into different size fractions, such as PM_10_ and PM_2.5_, comprising particles with an aerodynamic diameter of less than 10 and 2.5 µm, respectively. The PM_2.5_ fraction includes ultrafine particles with a diameter of less than 100 nm. Particles with the size of 5 µm to 10 µm in diameter have been observed to deposit mainly in the tracheobronchial tree, whereas particles with a diameter less than approximately 2.5 µm reach the respiratory bronchioles and alveoli [4,5,6]. A smaller fraction of ultrafine particles may also translocate the alveolar epithelial layer [7,8,9]. Inhalation of particles may induce oxidative stress and inflammation, which cause chronic inflammatory responses locally in the lung but also other systemic effects involving multiple organs and extrapulmonary diseases such as atherosclerosis, cancer, and diabetes [6,7,9,10].

Chronic inflammation and oxidative stress are strongly associated with the development and exacerbation of asthma and chronic obstructive pulmonary disease (COPD) [11]. PM exposure may thereby have a more harmful impact on patients with pulmonary diseases [12]. COPD is a heterogeneous disease characterized by airflow limitation and persistent respiratory symptoms, including obstruction of the small airways, chronic inflammation, bronchitis, and emphysema [11]. Tobacco smoking is the most important causative factor for COPD. However, around half of all COPD cases worldwide today are estimated to be related to non-tobacco-related risk factors [13], including exposure to air pollution from biomass smoke and black carbon [14,15]. The World Health Organization (WHO) recently updated the air quality guidelines for PM_2.5_ and PM_10_ to reduce air pollution-related deaths [16]. These guidelines do, however, not consider variation in the chemical and physical properties of PM but rather assume that all components have an equivalent hazard. Toxicological studies have demonstrated that particle size, shape, and chemical composition are important determinants of toxicity, implying that it is insufficient to rely on PM_2.5_ concentrations alone to explain the biological effects of PM exposure [17]. Studies that link individual PM components to toxic outcomes are still rare, and further studies are needed to disentangle the contribution of these components, especially regarding nitrate and sulfate. Most in vitro exposure studies focus on investigating one type of PM component in a cell culture system using only a single cell line, such as the bronchial epithelial cell line BEAS-2B or the alveolar epithelial cell line A549 with exposures performed at one single time point, in general, 24 h.

The objective of this study was to assess individual PM components and compare the relative hazardous effects of carbonaceous particles (soot), ammonium nitrate, ammonium sulfate, and copper oxide (CuO), which are common components of PM_2.5_ in ambient air, reflecting contributions from primary combustion, secondary inorganic constituents, and non-exhaust emissions (NEE), such as brake wear, from vehicular traffic [16,18,19,20,21,22]. We performed a comparison study examining exposure to the different PM components on epithelial cells from different airway locations (bronchial and alveolar) to evaluate whether there were similar responses to particles or not, as well as including lung fibroblasts that are involved in remodeling processes and the more complex model precision-cut lung slices (PCLS). We evaluated cell cytotoxicity, metabolic activity, ROS formation, and inflammatory responses. Our results indicate that CuO and soot are more hazardous PM components than ammonium nitrate and ammonium sulfate, inducing cytotoxicity and that the immunomodulatory response differs between bronchial and alveolar epithelial cells.

## 2. Results

### 2.1. Effects of Particle Exposure on Cell Viability

**Figure 1:**
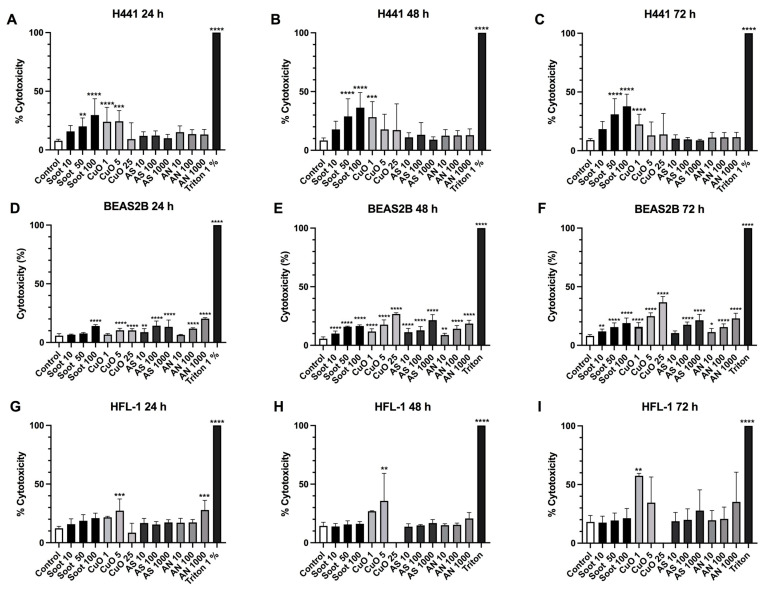
Cytotoxic effects of particle exposure. Measurement of cytotoxicity (% of positive control) analyzed by Lactate Dehydrogenase (LDH) released from H441 epithelial cells (**A**–**C**), bronchial epithelial cells BEAS-2B (**D**–**F**), and HFL-1 fibroblasts (**G**–**I**) and following exposure to soot (10, 50, and 100 µg/mL), copper oxide (CuO) (1, 5, and 25 µg/mL), ammonium sulfate (AS) (10, 100, and 1000 µg/mL) and ammonium nitrate (AN) (10, 100, and 1000 µg/mL) for 24 h (**A**,**D**,**G**), 48 h (**B**,**E**,**H**) or 72 h (**C**,**F**,**I**). 1% Triton-X100 was used as a positive control for cell death (100%). *n* = 11 for H441, *n* = 16 for BEAS-2B, and *n* = 7 for HFL-1. Data are representative of three independent experiments and presented as mean ± SD. One-way ANOVA followed by a post hoc Dunnett’s test for multiple comparisons was performed. Samples are compared to untreated control. * *p* < 0.05, ** *p* < 0.01, *** *p* < 0.001, **** *p* < 0.0001.

Soot exposure induced a concentration-dependent cytotoxic response over time (24–72 h) in the alveolar epithelial cells H441 (Figure 1A–C) and bronchial epithelial cells BEAS-2B (Figure 1D–F) but not in lung fibroblasts (Figure 1G–I). Most cytotoxic effect was observed in the alveolar epithelial cells following exposure to soot 100 μg/mL.

**Figure 2:**
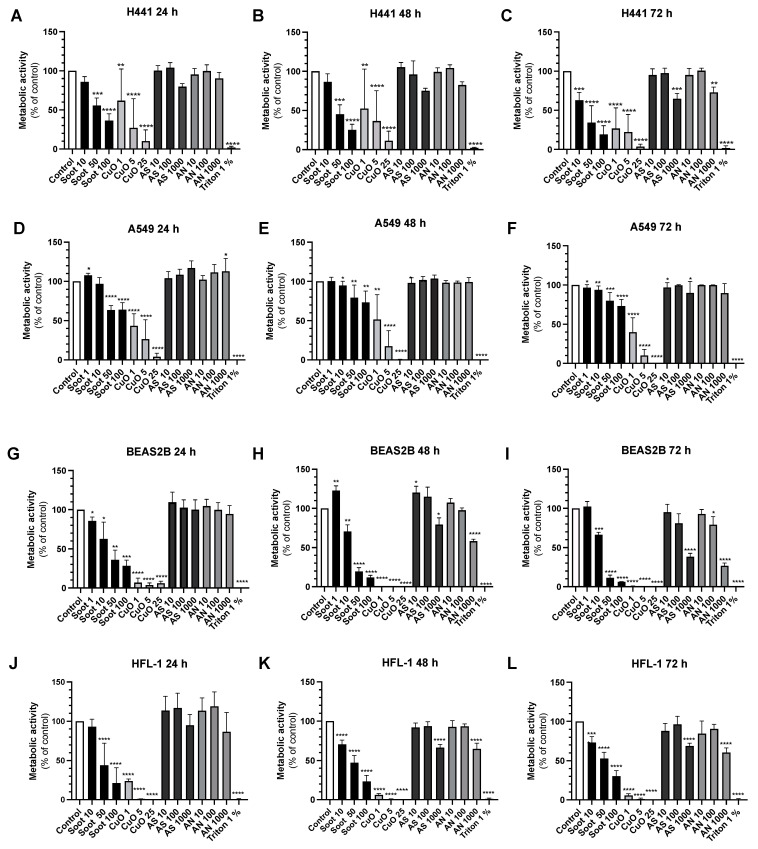
Effects of particles on cell viability. Changes in metabolic activity (% of control) were analyzed with a Tetrazolium salt (WST-1) assay in H441 epithelial cells, A549 alveolar epithelial cells, bronchial epithelial cells BEAS-2B, and lung fibroblasts (HFL-1) following exposure to either ammonium nitrate (AN) (10, 100, and 1000 µg/mL), ammonium sulfate (AS) (10, 100, and 1000 µg/mL), soot (1, 10, 50, and 100 µg/mL), copper oxide (CuO) (1, 5, and 25 µg/mL) for 24 h (**A**,**D**,**G**,**J**), 48 h (**B**,**E**,**H**,**K**) or 72 h (**C**,**F**,**I**,**L**) compared to untreated controls. 1% Triton-X100 was used as a positive control for cell death. *n* = 11 for H441 and *n* = 7 for HFL-1 and *n* = 4 for BEAS-2B at all exposures. Data are representative of three independent experiments with technical replicates. Data are presented as mean ± SD. One-sample *t*-tests with controls set at 100% were performed. * *p* < 0.05, ** *p* < 0.01, *** *p* < 0.001, **** *p* < 0.0001.

Metabolic activity was significantly reduced by soot exposure in a concentration-dependent manner at all time points in both alveolar and bronchial epithelial cells and lung fibroblasts (Figure 2A–L). The decrease in metabolic activity was more pronounced in H441 compared to A549 (Figure 2A–F). CuO induced cytotoxicity in a concentration and time-dependent manner (*p* < 0.0001) in the bronchial epithelial cells (Figure 1D–F) and induced cytotoxicity at all time points in alveolar epithelial cells (*p* < 0.01) (Figure 1A-C) and human lung fibroblasts (*p* < 0.01) (Figure 1G–I).

CuO, in a concentration-dependent manner, significantly reduced metabolic activity and viability in the different cell types at all time points, with the most pronounced effects in BEAS2B and HFL-1 (Figure 2G–L). AN and AS induced concentration-dependent cytotoxicity in bronchial epithelial cells (Figure 1D–F) but not in alveolar epithelial cells (Figure 1A–C) or in lung fibroblasts (Figure 1G–I), except for AN 1000 µg/mL at time point 24 h (*p* < 0.001) (Figure 1G). In line with these data, AN and AS, in general, did not show any effects on metabolic activity, except at the highest concentration (1000 μg/mL), which significantly reduced metabolic activity in lung fibroblasts and bronchial epithelial cells after 48 and 72 h and in alveolar epithelial cells at 72 h (Figure 2A–L).

**Figure 3:**
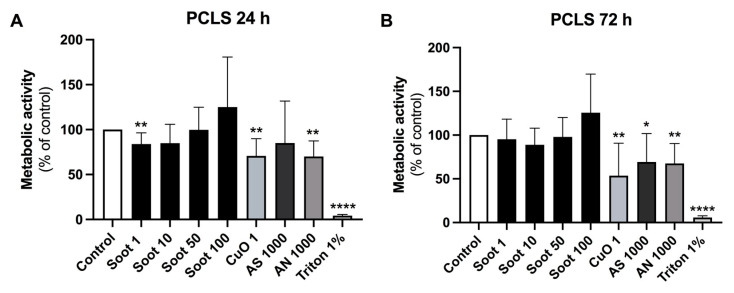
Effects of particles on cell viability in precision-cut lung slices. Measurement of metabolic activity (% of control) in rat precision-cut lung slices (PCLS) following exposure to copper oxide (CuO) (1 µg/mL), soot (1, 10, 50, and 100 µg/mL), ammonium sulfate (AS, 1000 µg/mL) and ammonium nitrate (AN, 1000 µg/mL) for 24 h (**A**) and 72 h (**B**). PCLS were obtained from four rat lungs with two technical replicates in each exposure setting (in total *n* = 8 slices in each group). Data are presented as mean ± SD. One-sample *t*-tests with controls set at 100% were performed. * *p* < 0.05, ** *p* < 0.01, **** *p* < 0.0001.

Exposure of PCLS to soot (1 µg/mL), CuO (1 μg/mL), or AN (1000 µg/mL) significantly reduced metabolic activity (*p* < 0.01), whereas soot (10 µg/mL) showed a tendency of reduced viability (*p* = 0.082) after 24 h (Figure 3A). After 72 h, metabolic activity was significantly reduced after exposure to CuO (1 μg/mL; *p* < 0.01), AS (1000 µg/mL; *p* < 0.05), or AN (1000 µg/mL; *p* < 0.01), whereas there were no significant alterations to soot exposures (Figure 3B).

**Figure 4:**
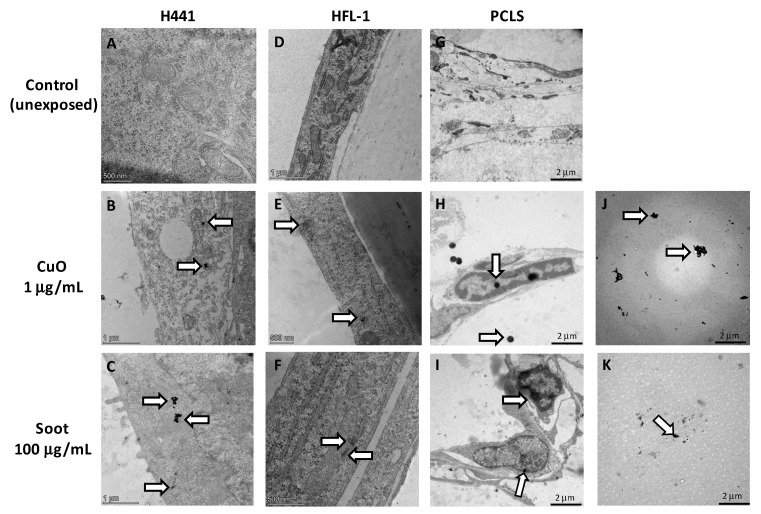
Transmission Electron Microscopy (TEM) images of particles and cellular uptake. TEM images of unexposed epithelial cells (H441) (**A**), fibroblasts (HFL-1) (**D**), and PCLS (**G**). TEM images of CuO particles at a concentration of 1 µg/mL and soot particles at a concentration of 100 µg/mL in exposed cells cultured on inserts, H441 (**B**,**C**) and HFL-1 (**E**,**F**). Arrows indicate particles located close to mitochondria with altered structure in cells exposed to CuO (**B**,**E**) and soot (**C**,**F**). Exposed precision-cut lung slices (PCLS) (**H**,**I**) with arrows indicating the particles. TEM images of CuO particles at a concentration of 1 µg/mL and soot particles at a concentration of 100 µg/mL in suspension without any cells (**J**,**K**).

Further analyses using transmission electron microscopy (TEM) (Figure 4) indicated that the particles were taken up by the alveolar epithelial cells (Figure 4B,C) and lung fibroblasts (Figure 4E,F) during 24 h of exposure. Soot and CuO particles were located deeper in the PCLS and not only located on the surface of the slice (Figure 4H,I). CuO particles were distributed as single particles within the cells and PCLS, whereas some aggregations of soot were observed, as also displayed in the TEM images on both the CuO and soot particles without any cells present (Figure 4J,K).

We did not observe any specific compartmentalization in the cells, such as in the lysosomes, but particles were found to be located close to mitochondria in both epithelial cells and fibroblasts. CuO 1 µg/mL and soot 100 µg/mL induced morphological changes, especially in mitochondria with alterations to internal structures (cristae) in the epithelial cells (Figure 4B,C) and in the fibroblasts (Figure 4E,F), which supports the obtained data on reduced metabolic activity (Figure 2) linked to mitochondria dysfunction.

**Figure 5:**
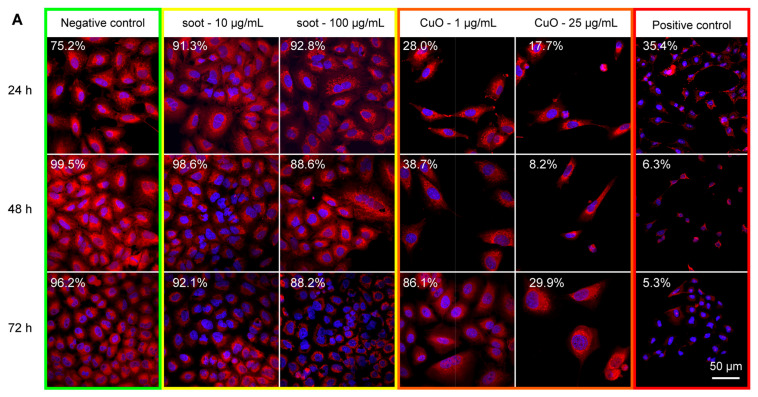

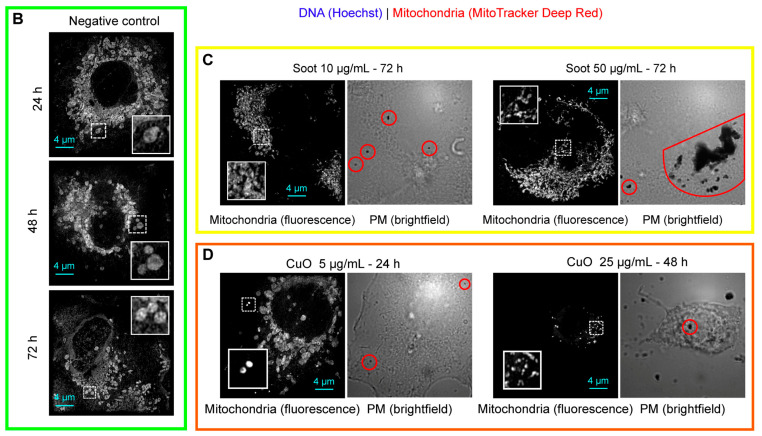
Fluorescence micrographs of A549 cells exposed to CuO and soot particles. Confocal Laser Scanning Microscope (CLSM) images of cells stained for DNA (blue) and mitochondria (red) indicated that exposure to Copper Oxide (CuO) significantly reduced cell numbers after 24, 48, and 72 h compared to the negative control (untreated cells) (**A**). Positive control of cells treated with 1% Triton-X100 is also shown for comparison. Measured cell confluency values are shown in the top left-hand corner of each image. Super-resolution structured illumination microscopy (SIM) images of cells stained for mitochondria indicated the effect of particle exposure on mitochondrial morphology (**B**–**D**). In untreated cells, mitochondria had a characteristic rounded shape (**B**). Following exposure to soot particles (highlighted with red outlines), mitochondria presented as small bright puncta, illustrative of mitochondrial shrinkage (**C**). The same effect was more pronounced following exposure to CuO particles (**D**).

Fluorescence microscopy images on A549 cells provided further insights into these results (Figure 5). Exposure to CuO 1 μg/mL and 25 μg/mL significantly reduced both the number density and confluency of A549 cells at all time points. However, no significant differences were observed when cells were unexposed (negative control) or exposed to soot particles 10 μg/mL and 100 μg/mL at the different time points (Figure 5A). Structured illumination microscopy of A549 cells stained with MitoTracker Deep Red was used to investigate the effect of particulate exposure on mitochondrial morphology. In negative control conditions (no particulate exposure) mitochondria in A549 cells had a characteristic rounded shape (Figure 5B). This morphology was disrupted, and small bright puncta, consistent with mitochondrial damage or degradation, were visible in cells exposed to higher concentrations of CuO (5 and 25 μg/mL) (Figure 5D). Similar mitochondrial disruption was also visible in cells exposed to higher concentrations of soot (50 μg/mL) (Figure 5C).

### 2.2. Exposure to Soot and CuO Induces Generation of ROS

**Figure 6:**
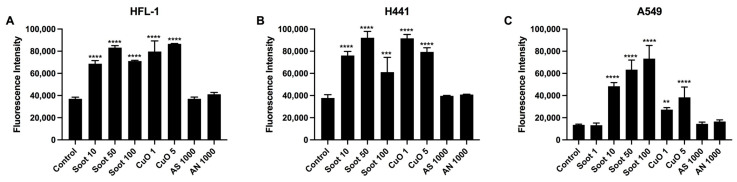
Effects of particle exposure on reactive oxidative species (ROS) formation in lung fibroblasts and epithelial cells. HFL-1 fibroblasts (**A**), (*n* = 3) and epithelial cells H441 (**B**), (*n* = 3) and A549 (**C**), (*n* = 6) labeled with DCFDA and exposed to soot (1, 10, 50, and 100 µg/mL), copper oxide (CuO) (1 and 5 µg/mL), ammonium nitrate (AN) (1000 µg/mL), or ammonium sulfate (AS) (1000 µg/mL) for 5 h. Cells were analyzed on a fluorescence plate reader at gain 650. Data are presented as mean ± SD. Statistical analysis was performed with one-way ANOVA followed by a post hoc Dunnett’s test for multiple comparisons vs. untreated control. ** *p* < 0.01, *** *p* < 0.001, **** *p* < 0.0001.

To evaluate the effect of particle exposure and if there were differences in reactive oxidative species (ROS) formation between the different cell types, 2′,7′-dichloroflourescein (DCF) fluorescence intensity was measured in lung fibroblasts and the alveolar epithelial cells H441 and A549 after 5 h of exposure to soot 1, 10, 50, and 100 µg/mL, CuO 1 and 5 µg/mL, AN 1000 µg/mL, and AS 1000 µg/mL. A concentration-dependent increase in ROS formation was observed for both soot and CuO in the exposed cells, where A549 cells showed less ROS formation compared to H441 (Figure 6A–C). Lower intensity was observed for the highest concentration of soot (100 µg/mL) in lung fibroblasts and H441 and CuO (5 µg/mL) in H441, which may be due to the adverse effect induced by these concentrations on viability. No noticeable generation of ROS could be observed for AN and AS when compared to the non-exposed control (Figure 6A–C).

### 2.3. Soot, CuO, Ammonium Sulfate, and Ammonium Nitrate Alter Inflammatory Responses

**Figure 7:**
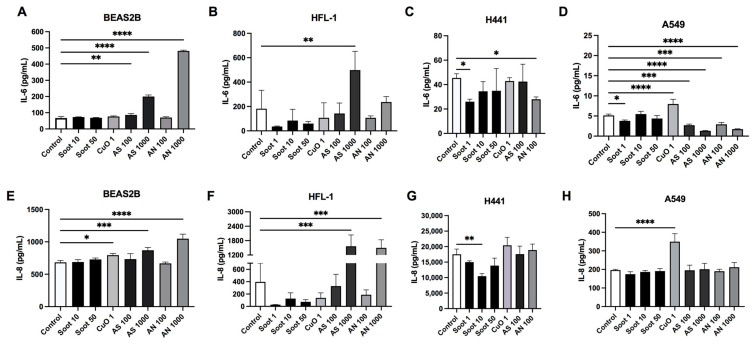

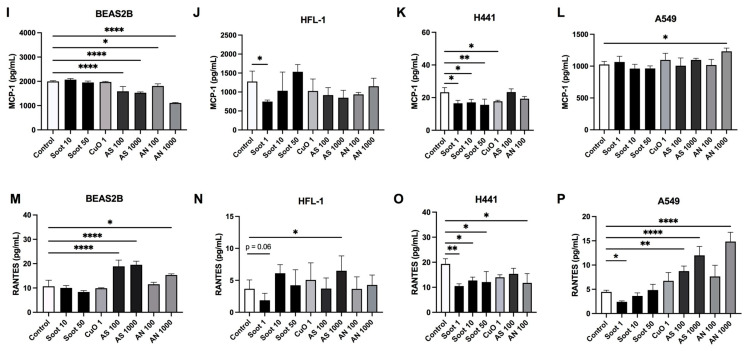
Effects of particle exposure on inflammatory mediators. Release of the following inflammatory mediators: interleukin (IL)-6 (**A**–**D**), IL-8 (**E**–**H**), Monocyte chemoattractant protein-1 (MCP-1) (**I**–**L**), and RANTES (**M**–**P**) were measured by multiplexed immunoassay analysis. BEAS-2B bronchial epithelial cells (**A**,**E**,**I**,**M**), lung fibroblasts HFL-1 (**B**,**F**,**J**,**N**), H441 alveolar epithelial cells (**C**,**G**,**K**,**O**), and A549 alveolar epithelial cells (**D**,**H**,**L**,**P**) were exposed to either soot (1, 10, 50, or 100 µg/mL), copper oxide (CuO; 1 µg/mL), ammonium sulfate (AS; 100 µg/mL or 1000 µg/mL), or ammonium nitrate (AN; 100 µg/mL or 1000 µg/mL) for 72 h. *n* = 3–4 individual experiments for each cell type and exposure. Data are presented as mean ± SD. Statistical analysis was performed with one-way repeated measurements (RM) ANOVA followed by a post hoc test Dunnett’s for multiple comparisons vs. untreated control. * *p* < 0.05, ** *p* < 0.01, *** *p* < 0.001, **** *p* < 0.0001.

The epithelial cells had different profiles in the secretions of inflammatory mediators (Figure 7). The bronchial BEAS-2B cells and alveolar A549 epithelial cells released higher amounts of monocyte chemoattractant protein (MCP)-1 (Figure 7I,L) compared to the alveolar epithelial cells H441 (Figure 7K). H441 released higher amounts of interleukin (IL)-6 (Figure 7C) and very high amounts of IL-8 (Figure 7G) compared to A549 cells (Figure 7D,H). We focused the inflammatory profile analyses on the lower concentrations of CuO and soot as higher concentrations of CuO (5 and 25 µg/mL) and soot (100 µg/mL) induced a higher degree of cytotoxicity and reduced the viability in the cell cultures. Exposures to AN 1000 µg/mL and AS 1000 µg/mL were not measured in H441.

Soot, in general, did not affect the release of inflammatory mediators from BEAS-2B (Figure 7). In alveolar epithelial cells, exposure to soot induced more alterations in H441 compared to A549. Only the soot concentration of 1 µg/mL significantly reduced the release of IL-6 and RANTES (Figure 7D,P) in A549, whereas in H441, soot 1 µg/mL reduced the release of IL-6, MCP-1 and RANTES (Figure 7C,K,O); soot 10 µg/mL reduced IL-8, MCP-1, and RANTES (Figure 7G,K,O); and soot 50 µg/mL reduced MCP-1 and RANTES (Figure 7K,O). In HFL-1, soot 1 µg/mL significantly reduced the release of MCP-1 and RANTES (Figure 7J,N).

CuO 1 µg/mL significantly increased the release of IL-6 in A549 (Figure 7D) and IL-8 in BEAS-2B (Figure 7E) and A549 (Figure 7H). CuO did not significantly affect the release of inflammatory mediators from H441 and HFL-1 (Figure 7).

AS 100 µg/mL significantly increased the release of IL-6 and RANTES (Figure 7A,M) and reduced MCP-1 (Figure 7I) in BEAS-2B, whereas in A549, IL-6 was significantly reduced (Figure 7D) and RANTES increased (Figure 7P). AS 1000 µg/mL significantly increased the release of IL-6, IL-8, and RANTES in both BEAS-2B (Figure 7A,E,M) and in HFL-1 (Figure 7B,F,N), whereas MCP-1 levels were reduced in BEAS-2B (Figure 7I) and IL-6 levels were reduced in A549 (Figure 7D).

Exposure to AN 100 µg/mL significantly reduced the release of MCP-1 in BEAS-2B (Figure 7I) and reduced the release of IL-6 in H441 (Figure 7C) and A549 (Figure 7D). AN 1000 µg/mL significantly increased the release of IL-6, IL-8, and RANTES (Figure 7A, E, and M) in BEAS-2B, increased the release of IL-8 in HFL-1 (Figure 7F) and increased MCP-1 and RANTES (Figure 7L and P) in A549, whereas MCP-1 was reduced in BEAS-2B (Figure 7I) and IL-6 was reduced in A549 (Figure 7D).

### 2.4. Effects of Particle Exposure on Release of Growth Factors

**Figure 8:**
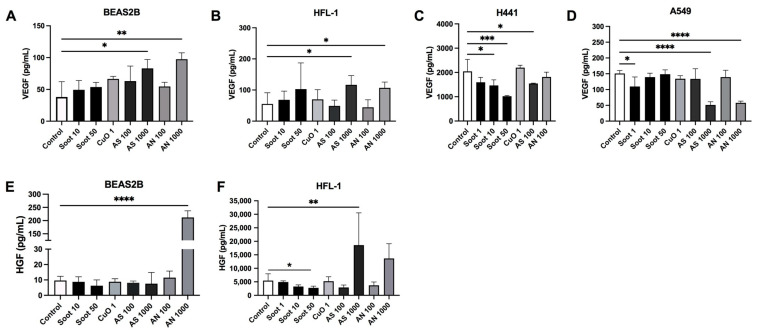
Effects of particle exposure on growth factors. The release of growth factors vascular endothelial growth factor (VEGF) (**A–D**) and hepatocyte growth factor (HGF) (**E,F**) were measured by multiplexed immunoassay analysis. Bronchial epithelial cells BEAS2B (**A**,**E**), lung fibroblasts HFL-1 (**B**,**F**), alveolar epithelial cells H441 (**C**) and A549 (**D**) were exposed to either (1, 10, 50, or 100 µg/mL), copper oxide (CuO; 1 µg/mL), ammonium sulfate (AS; 100 and 1000 µg/mL) or ammonium nitrate (AN; 100 and 1000 µg/mL) for 72 h. *n* = 3–5 for each cell type and exposure. Data are presented as mean ± SD. Statistical analysis was performed with one-way ANOVA followed by a post hoc test Dunnett’s for multiple comparisons vs. untreated control. * *p* < 0.05, ** *p* < 0.01, *** *p* < 0.001, **** *p* < 0.0001.

The release of growth factors differed among the epithelial cells, with high amounts of vascular endothelial growth factor (VEGF) being produced by H441 (Figure 8C) compared to BEAS-2B and A549 (Figure 8A and D). Lung fibroblasts released higher amounts of hepatocyte growth factor (HGF) (Figure 8F) than the epithelial cells. HGF was not measurable in A549 cells and below the recommended detection limits in H441.

Exposure to soot 10 and 50 µg/mL significantly reduced VEGF in H441 (Figure 8C) and soot 1 µg/mL significantly reduced VEGF in A549 (Figure 8D). AS 1000 µg/mL and AN 1000 µg/mL significantly reduced VEGF in A549 (Figure 8D), whereas VEGF levels were significantly increased in BEAS-2B (Figure 8A) and HFL-1 (Figure 8B). The release of HGF was significantly reduced at soot 50 µg/mL (Figure 8F) in HFL-1, whereas AS 1000 μg/mL increased HGF in HFL-1 (Figure 8F) and AN 1000 μg/mL increased HGF in BEAS2B (Figure 8E). CuO 1 µg/mL did not show any significant effects in either epithelial cells or fibroblasts (Figure 8A–F).

## 3. Discussion

We evaluated the relative hazard effects of PM components: elemental carbon (EC) and organic carbon (OC) (soot), CuO, ammonium nitrate (AN), and ammonium sulfate (AS) on bronchial and alveolar epithelial cells and lung fibroblasts, and briefly in a more advanced precision-cut lung slice (PCLS) model, through the evaluation of a range of immunotoxicity, viability, and metabolic endpoints. Our results indicated significant differences in the cytotoxicity of PM components to epithelial cells and fibroblasts, including differences in inflammatory mediator responses. Following exposure to soot particles, we observed a concentration-dependent increase in cytotoxicity in epithelial cells, whereas a concentration-dependent decrease in metabolic activity was observed in both epithelial cells and fibroblasts. AN and AS induced negligible cytotoxicity and did not alter metabolic activity, except at very high concentrations, after 72 h. We further investigated if the particles increased ROS formation and if there were differences in ROS generation between the different cell types. CuO and soot significantly increased intracellular ROS formation in both alveolar epithelial cells and fibroblasts, whereas AS and AN did not. The alveolar cell line H441 responded with more ROS formation and alterations in cytokine release than A549. In previous in vitro studies, soot particles have been shown to induce mitochondrial damage and increased generation of ROS [23,24,25]. In a previous study in bronchial epithelial cells and in line with our data, soot exposure reduced viability in a dose-dependent manner with a significant reduction at higher concentrations of soot, as well as a reduced mRNA expression of superoxide dismutase (*SOD2*) and nuclear factor erythroid-2-related factor 2 (*NFE2L2/nrf2*) [24]. Both *SOD2* and *nrf2* are involved in antioxidant protection, and the downregulation of *nrf2* has been linked to the initiation and progression of COPD [26]. In a recent in vitro study with human bronchial epithelial cells, a secondary organic coating of the soot particles was found to further increase cytotoxicity [27].

AN and AS have been shown to be important secondary inorganic fractions of PM_2.5_ associated with health impacts [17,28]. Consistent with previous studies, we did not observe any cytotoxicity associated with exposure of epithelial cells and fibroblasts to AN or AS [17]. However, in the present study very high concentrations of AS and AN induced immunomodulatory effects in epithelial cells and fibroblasts with a pronounced increase in levels of the pro-inflammatory cytokines IL-6 and IL-8 and the growth factors VEGF and HGF, markers that relate to airway inflammation and remodeling in asthma and COPD [11]. Previous in vivo studies with mice models indicated that AS increased asthmatic and inflammatory responses [29] and that chronic exposure to nitrate ions induced airflow obstruction [30].

In the present study, we compared the effect of the different PM_2.5_ particles soot, AN, and AS with the effect of the metal oxide CuO. Cu is currently a component of brake wear and can be regarded as a proxy for NEE sources found in PM_2.5_. We found that CuO induced high levels of ROS and reduced metabolic activity. Previous studies in A549 epithelial cells have shown that CuO nanoparticles were the most potent metal oxide in regards to DNA damage and cytotoxicity compared to other nanoparticles, including zinc and iron [24]. CuO nanoparticles have previously been shown to induce cytotoxicity, mutagenicity, oxidative stress, and mitochondrial impairment [31,32]. Our microscopy images revealed that the CuO particles were localized near mitochondria and that the mitochondria displayed phenotypic alterations. CuO is known to be taken up by mitochondria and involved in mitochondrial respiration [33]. Exposure to higher concentrations of CuO has been shown to increase the production of ROS, and ROS are known to cause mitochondrial damage and disruption of the mitochondrial respiratory chain [34]. In an in vitro study on HepG2 cells, CuO nanoparticles induced apoptosis via interactions with the mitochondrial pathway [25].

Our results indicated that exposure to soot, CuO, AN, and AS induces different immunomodulatory responses in airway structural cells. Fibroblasts are thought to be important immune regulators and are involved in the persistence of chronic inflammation [35]. One hypothesis for the disparity is that the epithelial cells respond with a faster inflammatory response to particles, while the fibroblasts have a slower but more prolonged response. In the present study, soot reduced the release of inflammatory cytokines in alveolar epithelial cells and in fibroblasts but showed no significant effects in bronchial epithelial cells, whereas AS and AN significantly increased the release of IL-6, IL-8, and RANTES in bronchial epithelial cells and fibroblasts. Increased levels of IL-6 and IL-8 have previously been observed after soot exposure in bronchial epithelial cells [36,37]. In contrast, the alveolar epithelial cells H441 and A549 responded with a reduced release of IL-6 to both soot and AN exposure in the present study. The H441 cells, in comparison to A549 and BEAS-2B, produced high amounts of VEGF, an important growth factor for angiogenesis, which was reduced after soot exposure. A previous study in an alveolar–capillary co-culture lung model showed that exposure to PM_2.5_ and black carbon reduced the angiogenic ability of endothelial cells to form new blood vessels [38]. HGF is involved in the proliferation of epithelial and endothelial cells and interacts with VEGF in repair processes in the lung [39]. In the present study, exposure to soot reduced the release of HGF in fibroblasts. Reduced levels of HGF and VEGF may result in fewer capillaries and a reduced gas exchange capacity in the lung, which has been linked to the development of emphysema [39,40]. These data indicated that it is important to study cellular effects both in alveolar and bronchial regions as the inflammatory responses in the bronchial and alveolar regions differ, which can be linked mechanistically to the evolution of emphysema, peribronchial fibrosis, and obstructions observed in COPD.

In the current study, we used well-characterized materials, measuring responses over a range of doses and time points with multiple cell lines that are consistent with those used for high throughput screening methodologies. Toxicity studies are usually performed in a single cell culture model during a 24-h exposure, and later time points are often absent in the literature. Cytotoxic and inflammatory responses are expected to vary between different cell types, but differences may also be observed within a specific cell type depending on the origin of the specific cell line or primary cells and their location (bronchial or alveolar). To capture a wider range of toxic responses, we increased the exposure time to 72 h and incorporated different airway structural cells, e.g., bronchial and alveolar epithelial cells, fibroblasts, and the more advanced ex vivo model PCLS. However, additional studies with PCLS and using primary cells are important to further establish the in vitro to in vivo coherence of these results. Future studies should also include co-cultures with immune cells, to address cellular crosstalk and influences of compositional variable PMs on macrophages and dendritic cells. In the present study, we investigated the core fraction of soot to observe what the effect of uncoated soot is on different airway structural cells and not secondary organic soot/aged soot, which is more common in PM samples [20]. Soot coated with organics appears to give more toxic responses compared to uncoated soot [27] and the soluble organic fraction has been linked to cardiovascular effects [41,42]. In the current study, we exposed the cells and PCLS to high concentrations of the particles, as AS and AN are soluble constituents of ambient PM, to observe if there were concentration and potential pro-inflammatory/cytotoxic responses, even at very high, non-environmentally relevant concentrations (1000 µg/mL). This study demonstrated, consistent with previous observations, that AS and AN are less hazardous PM constituents compared to CuO and soot related to cytotoxicity. This is true even though the relative dose of the mass added per solute volume that reaches the cell surface will be higher for the non-soluble particles compared to the soluble entities (AN and AS) as particles tend to diffuse or aggregate and sediment to the cell surface, contrary to the fully soluble compounds. This is an important observation given the contribution of secondary inorganics to real-world PM_2.5_ and highlights that not all PM components have an equal hazard.

## 4. Methods

### 4.1. Preparation of Particles

Ammonium nitrate (AN), ammonium sulfate (AS), copper oxide (CuO), and soot were analyzed (see Table 1). AN (99.99%) was purchased from Sigma-Aldrich (Sigma-Aldrich, Saint Louis, MO, USA) and AS (99.5%) was purchased from Acros Organics (Thermo Fisher Scientific, Waltham, MA, USA). Copper (II) oxide (CuO) powder comprising particles smaller than 50 nm in diameter was purchased from Sigma-Aldrich (Sigma-Aldrich, Saint Louis, MO, USA).

Uncoated soot particles were produced using a miniCAST 5201 Type BC generator (Jing Ltd., Zollikofen, Switzerland) operated in a premixed flame mode, where air was added to propane fuel [21,43]. A set point was chosen to yield particles with a high elemental carbon (EC) content. The geometric mean diameter of the soot particles was 88 nm ± 4 nm, measured by a scanning mobility particle sizer (SMPS) (Electrostatic Classifier Series 3080 and CPC 3776 low flow, TSI Incorporated, Shoreview, MN, USA) [43,44]. Before being sampled on membrane filters (Nuclepore Track-Etched Membranes, Global Life Sciences Solutions USA LLC, Marlborough, MA, USA), the aerosol was dried using a diffusion dryer [43]. The soot particles were then scraped off the filters with a spatula and stored in powdered form. To prepare the stock solutions, the particles were suspended in either double distilled water (soot, AN, and AS) or cell culture medium with 10% serum (CuO) based on previous studies [17,23,36]. The suspensions were then vortexed (Janke & Kunkel VF2, IKA, Staufen im Breisgau, Germany) vigorously for 20 s, sonicated twice (Bransonic 220, Kebo-Grave, Stockholm, Sweden) for 20 s and then vortexed for a further 20 s. Prior to each experiment, the suspensions were vortexed for 10 s. The stock solutions were further diluted in the relevant cell culture medium to achieve the desired final concentration. Concentrations of particles used for the cell exposures were based on previous studies on CuO [23,31,45,46,47], soot [36,48], and AS and AN [17].

### 4.2. Content of Organic and Elemental Carbon in the Soot Particles

The organic carbon (OC) and elemental carbon (EC) content of the suspended soot particles was determined by thermal–optical analysis (TOA), according to the EUSAAR_2 protocol [49]. The stock solution was diluted to three different concentrations within the optimal measurement range of the instrument. The concentration of the stock solution was calculated as 5.2 mg/mL compared to the nominal value of 5 mg/mL. EC accounted for ~85% of the total carbon (thus, OC ~15% of the total carbon).

### 4.3. Epithelial Cell Cultures and Lung Fibroblasts

For epithelial cell exposures and comparison, we used the human distal airway/alveolar cell lines NCl-H441 and A549 and the human bronchial epithelial cell line BEAS-2B, all obtained from ATCC, Manassas, VA, USA. NCl-H441 is an adenocarcinoma cell line derived from a human distal lung epithelial origin with the capacity to form monolayers with barrier properties resembling human alveolar epithelial cells characteristic of both type II and club cells [50]. A549 is obtained from a human pulmonary adenocarcinoma and is representative of alveolar type II cells due to the presence of surfactant proteins and lamellar bodies [51]. BEAS-2B is a non-tumorigenic cell line established from normal bronchial epithelium. NCI-H441 and BEAS-2B were cultured in RPMI 1640 medium (Thermo Fisher Scientific, Waltham, MA, USA), supplemented with 10% FCIII, 1% antibiotic, and anti-mycotic solution (AB/AM, Sigma-Aldrich), and 1 mM pyruvate (Thermo Fisher Scientific, Waltham, MA, USA) in an incubator at 37 °C and 5% CO_2_. H441 cells were obtained in passage 54 and used in passages 56–62. The cell line A549 was cultured in DMEM medium (Thermo Fisher Scientific, Waltham, MA, USA), supplemented with 10% FCIII, 1% antibiotic and antimycotic solution (AB/AM, Sigma-Aldrich), and 1 mM pyruvate (Thermo Fisher Scientific, Waltham, MA, USA) at 37 °C and 5% CO_2_ and used in passages 4–10. The cell line human fetal lung fibroblasts (HFL-1) (CCL-153, ATCC, Manassas, VA, USA) were cultured in 75 cm^2^ cell culture flasks (Sarstedt, Nümbrecht, Germany) in Dulbecco’s modified Eagle medium (DMEM) (Thermo Fisher Scientific, Waltham, MA, USA) supplemented with 10% fetal clone serum (FCIII) (Thermo Fisher Scientific, Waltham, MA, USA), 0.5% gentamicin, 1% amphotericin B, and 1% L-glutamine (all from Gibco BRL, Paisley, UK) in an incubator at 37 °C and 10% CO_2_. HFL-1 cells were obtained in passage 13 and used in passages 15–21. The cells were cultured in DMEM supplemented with 0.4% FCIII serum to avoid interference with components in the serum during exposure studies. In experiments examining the production of ROS and LDH release, DMEM without phenol red and pyruvate (Thermo Fisher Scientific, Waltham, MA, USA) was used in order to avoid interference when measuring fluorescence.

### 4.4. Precision-Cut Lung Slices

Precision-cut lung slices (PCLS) were generated as previously described [52,53]. from 19–21-week-old male Sprague Dawley rats (540–680 g; Janvier, France) kept under controlled conditions (22 °C, 55% humidity, 12-h day/night rhythm and food and water ad libitum). The animal experiments were approved by the regional animal experiment ethical review board in Malmö/Lund (Dnr 5.8.18-09470/2021). Briefly, rats were euthanized with an i.p. dose (150 mg/kg) of pentobarbital. Twenty-five milliliters of 1% pre-warmed low-melting agarose (Sigma-Aldrich) mixed with medium were instilled into the lungs through an attached tracheal cannula. The thorax was opened, and the heart–lung package was removed and put on ice to solidify the agarose. The lung lobes were cut into 1 cm cylinder cores, which were further cut into 350 μm slices using a Krumdieck tissue slicer (Alabama Research and Development, Munford, AL, USA) filled with ice-cold DMEM/F12 with 1% (*v/v*) AB/AM solution. Slices were then immediately collected and placed in a petri dish with a complete culture medium: DMEM/F12, supplemented with 1% (*v/v*) AB/AM, 1% pyruvate, and 1% (*v/v*) Insulin–Transferrin–Selenium (ITS-G, Gibco, Thermo Fisher Scientific), in a humidified incubator (37 °C and 5% CO_2_). The medium was changed every half hour for the first two hours and then every hour for the next two hours to remove agarose and to avoid the accumulation of inflammatory mediators and cell debris as the cutting procedure induced some cell damage, which may interfere with analyses. Slices were then stored overnight before experiments were performed. The medium was changed every day and before any experiments. Upon experiments, slices were moved to 24-well plates (one slice per well) with 1 mL of complete medium in each well.

### 4.5. Exposure of Cells and Lung Slices to Particles

Cells were seeded in 6-well plates (200,000 cells/well, surface area of the well: 9.6 cm^2^) and 2 mL culture medium (NUNC, Thermo Fisher Scientific, Waltham, MA, USA) or in 96-well plates (7000 cells/well, surface area of the well: 0.32 cm^2^ and 200 μL culture medium) (Sarstedt, Nümbrecht, Germany). Stock suspensions of particles were further diluted in cell medium at concentrations 10, 100, and 1000 µg/mL for AN and AS; 1, 10, 50, and 100 µg/mL for soot; and 1, 5, and 25 µg/mL for CuO. H441, A549, BEAS2B, HFL-1, or PCLS were then exposed to particles for 5 h, 24 h, 48 h, or 72 h, at 37 °C and 5% or 10% CO_2_, depending on cell type and experimental conditions. The soot and CuO concentrations 1, 5, 10, 25, 50, and 100 µg/mL, converted to mass per cell surface area, corresponded to 0.625, 3.125, 6.25, 31.25, and 62.5 μg/cm^2^ in the 96-well plates with 200 μL medium, based on that all particle sediments on the cells in the culture plate well.

### 4.6. Measurement of Cytotoxicity

For the assessment of cytotoxicity, we used a Cytotoxicity Detection Kit (Roche, Basel, Switzerland) based on the measurement of released lactate dehydrogenase (LDH). Cells were seeded in 96-well plates (7000 cells/well). After particle exposure for 24, 48, or 72 h, the cell medium was collected from H441, BEAS-2B, and HFL-1 and analyzed for LDH, according to the manufacturer’s instructions. One percent Triton-X100 was used as a positive control for necrotic cell death. Absorbance was measured with a microplate reader (Multiskan GO, Thermo Fisher Scientific, Waltham, MA, USA) at 492 nm with a reference wavelength of 620 nm. To determine the cytotoxicity (%), the following formula was used: Cytotoxicity (%) = (Exp. value—negative control)/(positive control—negative control).

### 4.7. Measurement of Metabolic Activity

Metabolic activity was analyzed using the Cell Proliferation Reagent WST-1 (Sigma-Aldrich, Saint Louis, MO, USA). Tetrazolium salt (WST-1) is cleaved to formazan by cellular mitochondrial dehydrogenases in viable cells. Thus, the amount of formazan produced is associated with the activity of mitochondrial dehydrogenases and correlates directly with the number of viable cells. Cells (H441, A549, BEAS-2B, and HFL-1) were seeded in 96-well plates (7000 cells/well). After particle exposure for 24, 48, or 72 h, the medium was exchanged and diluted WST-1 reagent (10x) in cell medium was added to the cells or lung slices and incubated at 37 °C for 1 h before measuring the absorbance with a microplate reader (Multiskan GO, Thermo Fisher Scientific, Waltham, MA, USA) at 440 nm with a reference wavelength of 620 nm, according to the manufacturer’s instructions. To calculate the metabolic activity as a percentage of control, the following formula was used: Metabolic Activity (% of control) = (Exp. value—negative control)/(positive control—negative control).

### 4.8. Measurement of Reactive Oxygen Species (ROS) Formation

Reactive oxygen species (ROS) were analyzed using the DCFDA/H2DCFA Cellular ROS assay kit (Abcam, Cambridge, UK), which is based on the oxidization of DCFDA to fluorescent DCF by ROS. Cells (HFL-1, H441, and A549) were seeded in a dark, clear, flat bottom 96-well microplate with 25,000 cells per well in 200 μL of culture media and left to adhere overnight. Following manufacturer’s instructions, the cells were incubated with DCFDA solution for 45 min and then treated with soot (1, 10, 50, and 100 µg/mL), CuO (1 and 5 µg/mL), AN (1000 µg/mL), and AS (1000 µg/mL) for 5 h. The plate was then analyzed using a CLARIOstar fluorescence reader (BMG Labtech, Ortenberg, Germany) at Ex/Em = 485/535 nm.

### 4.9. Measurements of Total Protein Concentration

The total protein concentration in the cell lysate was determined with a Pierce bicinchoninic acid (BCA) Protein Assay Kit (Thermo Fisher Scientific, Waltham, MA, USA) using bovine serum albumin (BSA) as standard. The assay was performed according to the manufacturer’s instructions, and absorbance was measured with a microplate reader (Multiskan GO, Thermo Fisher Scientific, Waltham, MA, USA) at 562 nm. BSA standard curves were plotted, and the protein concentration of the samples was calculated.

### 4.10. Fluorescence Microscopy

For fluorescence microscopy analysis, A549 cells were cultured in chambered coverslips (Ibidi GmbH, Munich, Germany). After exposure to particle suspensions and control solutions for 24, 48, and 72 h (as described above), cells were incubated with 2 µg/mL Hoechst 3342 (Thermo Fisher Scientific, UK) and 300 nM MitoTracker Deep Red (Thermo Fisher Scientific, UK) for 30 min and washed three times with PBS before fixation using 4% PFA. Diffraction-limited images were captured using a confocal laser scanning fluorescence microscope (CLSFM) (FV1000, Olympus, Tokyo, Japan) with a 60×/1.35 oil immersion objective lens with acquisition settings optimized for image quality. The cell confluency in each captured image was estimated using a median-filtered copy of the MitoTracker Deep Red image channel. Filtered images were binarized to estimate the fraction of the substrate covered by cells using a manually optimized pixel value threshold based on Otsu’s method. Super-resolution structured illumination microscopy (SIM) was performed using a custom-built microscope system [54] with a 60×/1.3 objective lens in which sinusoidal excitation patterns were generated by interfering with the first diffracted orders from a liquid crystal on silicon spatial light modulator illuminated with laser light at 405 nm (Hoechst) and 638 nm (MitoTracker Deep Red).

### 4.11. Transmission Electron Microscopy (TEM)

For TEM images, cells (HFL-1 and H441) were cultured on inserts (50,000 cells/wells) (Costar HTS Transwell-24 System (Individual Transwell inserts with 6.5 mm diameter membranes), Ref 3395, Corning Incorporated, Kennebunk, ME, USA) in 24-well plates. Cells and PCLS were exposed to CuO (1 μg/mL) and soot (10 and 100 μg/mL) for 24 h in corresponding cell culture media. The inserts with cells and PCLS were then fixed in 0.1 M Sorensen’s phosphate buffer, pH 7.4, 1.5% formaldehyde, and 1.5% glutaraldehyde at RT for 1 h. After fixation, the samples were washed three times in 0.1 M Sorensen’s phosphate buffer pH 7.4 and then dehydrated in a graded series of ethanol (50%, 70%, 80%, 90%, and twice in 100%). The samples were embedded in pure Polybed 812, and the polymerized block was sectioned with a Leica UC7 ultramicrotome (Leica Microsystems GmbH, Wetzlar, Germany). The sections were mounted on a pioloform-coated copper Maxtaform H5 grid and stained with 4% Uranyl acetate. Soot and CuO particles in DMEM cell culture media were directly added to the grids as small drops and stained with 4% Uranyl acetate. Images were acquired using a Tecnai BioTWIN transmission electron microscope (Field Electron and Ion (FEI) Company, Hillsboro, OR, USA).

### 4.12. Measurement of Immune Response with Multiplexed Immunoassay Analysis

Epithelial cells (BEAS-2B, H441, and A549) and fibroblasts (HFL-1) were seeded in 6-well plates (200,000 cells per well and 2 mL medium/well). At 80% confluency, the cells were exposed to the particle suspensions for 72 h. The exposure area in the 6-well plates corresponded for the concentrations 1, 10, 50, 100, and 1000 µg/mL to 0.21, 2.1, 10.4, 20.8, and 208 μg/cm^2^. The cell medium and lysate were then collected and stored at -80 °C for further analysis. Inflammatory mediators and growth factor levels in the cell culture supernatant were determined using multiplexed immunoassays from Bio-Rad (human cytokine screening panel) analyzed on a Luminex platform (Bio-Plex 200, Bio-Rad Life Science, Hercules, CA), according to the manufacturer’s instructions. The selected panel of cytokines and growth factors was based upon previous studies on human lung fibroblasts (HFL-1) [55] and bronchial epithelial cells (BEAS2-2B) [56], which included the common inflammatory mediators: interleukin (IL)-6, IL-8, monocyte chemotactic protein (MCP-1), and RANTES, and growth factors involved in remodeling; vascular endothelial growth factor (VEGF) and hepatocyte growth factor (HGF). Calibration curves were fitted using a five-point regression model, and the results were evaluated using Bio-Plex Manager Software 6.0 (Bio-Rad). The lower limits of quantification were 1.4 (IL-6), 3.0 (IL-8), 2.6 (MCP-1), 0.12 (RANTES), 3.9 (VEGF), and 7.6 (HGF) pg/mL, respectively.

### 4.13. Statistical Analysis

Data are presented as mean ± standard deviation (SD). All statistical analyses were performed using the software GraphPad Prism 9.5.1 (San Diego, CA, USA). A value of less than 0.05 (*p* < 0.05) was used as a threshold for statistical significance. These data were analyzed with one sample *t*-test or one-way ANOVA followed by the post hoc Dunnett’s multiple comparisons test to compare the samples against the untreated control.

## 5. Conclusions

In this study, clear differences in the hazard of particles and particle-associated chemicals in terms of their cyto- and immune-toxicological impacts on different structural cells in the airways were shown. Furthermore, we observed distinct differences in the degree of baseline responses between cell types, highlighting the importance of selecting a cell system that can reflect/measure the expected effect when performing toxicity studies. In general, the cytotoxic response was more pronounced in epithelial cells compared to fibroblasts. Soot exposure induced more immune suppression in alveolar cells and limited inflammatory responses in bronchial epithelial cells. Copper oxide was found to be highly toxic, resulting in low cell viability and a high generation of ROS, followed by soot and limited effects of ammonium nitrate and ammonium sulfate, indicating a low hazard for these inorganic particles as no effects were observed at concentrations likely to be within realistic exposure levels. Information about the differential toxicity of PM components can enable decision-makers to develop better evidence-based air quality regulations that are based on toxicology studies rather than mass concentrations.

Declaration of competing interest: The authors declare that they have no known competing financial interests or personal relationships that could have appeared to influence the work reported in this paper.

## Figures and Tables

**Table 1:** Size and shape of particles used in the experiments.

Particle	Size (Diameter/Mass)	Shape
CuO	<50 nm 79.545 g/mole (molecular weight)	Spherical
Airborne soot	88 nm	Fractal-like
Ammonium nitrate	80.04 g/mole (molecular weight)	Crystallizes in a rhombohedral crystal structure
Ammonium sulfate	132.14 g/mole (molecular weight)	Crystallizes in an orthorhombic crystal structure

## Data Availability

The datasets supporting the conclusions of this article are available upon request from the corresponding author.
